# Comparison of the optical coherence tomographic characters between acute Vogt-Koyanagi-Harada disease and acute central serous chorioretinopathy

**DOI:** 10.1186/1471-2415-14-87

**Published:** 2014-06-28

**Authors:** Dusheng Lin, Weiqi Chen, Guihua Zhang, Huichun Huang, Zhaotao Zhou, Lingping Cen, Haoyu Chen

**Affiliations:** 1 Joint Shantou International Eye Center, Shantou University & the Chinese University of Hong Kong, North Dongxia Road, 515041 Shantou, China

**Keywords:** Vogt-Koyanagi-Harada disease, Central serous chorioretinopathy, Spectral-domain optical coherence tomography

## Abstract

**Background:**

Acute Vogt-Koyanagi-Harada (VKH) disease and acute central serous chorioretinopathy (CSCR) are two common disorders with serous retinal detachment caused by dysfunction of choroid. The purpose of this study is to compare the morphological changes of these two diseases with spectral domain optical coherence tomography (SD-OCT).

**Methods:**

In this retrospective comparative study, the SD-OCT images of 65 eyes with acute VKH and 52 eyes with acute CSCR were reviewed for the presence of subretinal fluid, folds of retinal pigment epithelial (RPE), fluctuation of internal limiting membrane (ILM), subretinal septa, retinal pigment epithelium detachment (PED) and bulge of RPE. The foveal thickness was measured using the manual caliper of OCT software. The characteristics of SD-OCT were compared between two diseases.

**Results:**

Subretinal fluid was present in both diseases. Folds of RPE, fluctuation of ILM, subretinal septa were seen only in VKH. Bulge of RPE presented only in CSCR. PED was more common in CSCR than in VKH (44.2% vs 3.1%, p < 0.001). The thickness of fovea and RPE undulation index were significantly greater in VKH compared to that in CSCR (746.7 ± 423.8 vs 444.9 ± 158.8 μm, p < 0.001 and 1.0667 ± 0.0509 vs. 1.0177 ± 0.0023, p = 0.003).

**Conclusion:**

Our study showed that although VKH and CSCR share similar features on SDOCT, there are characteristic differences between both disease entities.

## Background

Vogt-Koyanagi-Harada (VKH) disease is a common cause of panuveitis involving both eyes among darkly pigmented races [1]. It accounts for about 8% to 13% of total uveitis patients in Eastern Asian [2]. The acute phase of VKH disease is characterized by diffuse choroidal inflammation and serous retinal detachment in the posterior pole [3]. Central serous chorioretinopathy (CSCR) is a common disease characterized by serous detachment of the neurosensory retina and/or the retinal pigment epithelium (RPE) that often involves the macula.

There are some similarities as well as differences between the features of VKH and CSCR. Both of them are caused by disorder of choroid and RPE. They also share some common clinical manifestation, such as serous retinal detachment, leakage from RPE on fundus fluorescein angiography (FFA) [4-9]. However, VKH is an inflammatory disease while CSCR occurs due to vascular compromise in choroid. The treatment of VKH and CSCR are opposite. High dose systemic corticosteroid therapy is required in VKH but forbidden in CSCR [3,10,11]. Failure in differentiating CSCR from VKH diseases may result in inappropriate use of corticosteroids, leading to exacerbation of disorder.

Spectral-Domain Optical coherence tomography (SD-OCT) provides non-invasive high speed, high resolution, three-dimensional cross-section imaging of macula. It is widely used for investigation and diagnosis of macular diseases [12,13]. In the present study, we investigated the features of VKH and CSCR on SD-OCT images. To our knowledge, there has not been a head-to-head comparison of OCT features for these two diseases.

## Methods

### Subjects

This is a retrospective comparative study. It was approved by the Institutional Review Board of Joint Shantou International Eye Center and complied with the Declaration of Helsinki. Informed consent was waived due to the retrospective design of this study.

The patient database of Joint Shantou International Eye Center, Shantou University & the Chinese University of Hong Kong from January 2011 to February 2013 was searched. The medical charts of patients diagnosed as VKH disease or CSCR received further review. The patients received comprehensive ophthalmic examinations. Best-corrected visual acuity (BCVA) was measured with the Chinese standard logarithm visual chart and converted into logarithm of the minimal angle of resolution (LogMAR) unit. Intraocular pressure was measured using a non-contact tonometer. Slit lamp biomicroscopy was performed to examine anterior segment and retina with mydriasis. Fundus fluorescein angiography (FFA) was performed with Heidelberg Retina Angiograph (HRA2, Heidelberg Engineering, Germany). None of the subjects had received systemic corticosteroid treatment before the OCT scan.

Diagnosis of VKH disease was based on the diagnostic criteria laid down by the International Committee in 2001 [1]. Diagnosis of acute CSCR was based on typical angiographic findings of focal neurosensory retinal detachment or retinal pigment epithelium (RPE) detachment with leakage at the level of the RPE seen on fluorescein angiography that resolved spontaneously [14]. Exclusion criteria were as follows: (1) the time from onset to OCT scan was more than four weeks; (2) coexistence of other retinal diseases; and (3) the image quality score of OCT was lower than 4/10 (signal strength).

### Spectral domain optical coherence tomography

**Figure 1:**
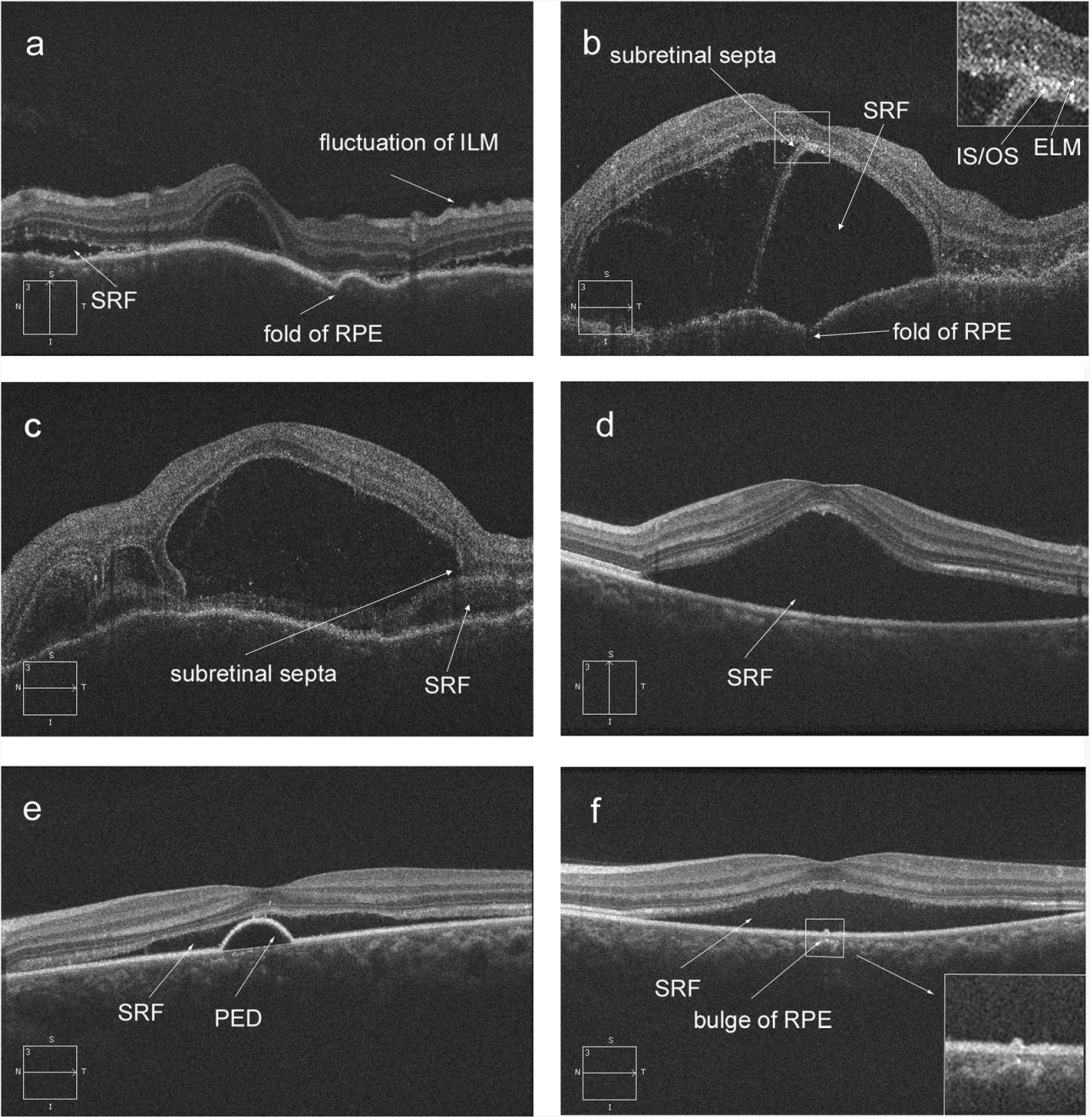
**Spectral-domain optical coherence tomography (OCT) features d of acute Vogt-Koyanagi-Harada (VKH) disease and acute central serous chorioretinopathy (CSCR).** Figure 1**a**,**b** and **c** showed the OCT images of VKH. Figure 1**d**,**e** and **f** showed the OCT images of CSCR. SRF = subretinal fluid; RPE = retinal pigment epithelium; PED = pigment epithelium detachment; ILM = inner limiting membrane.

**Figure 2:**
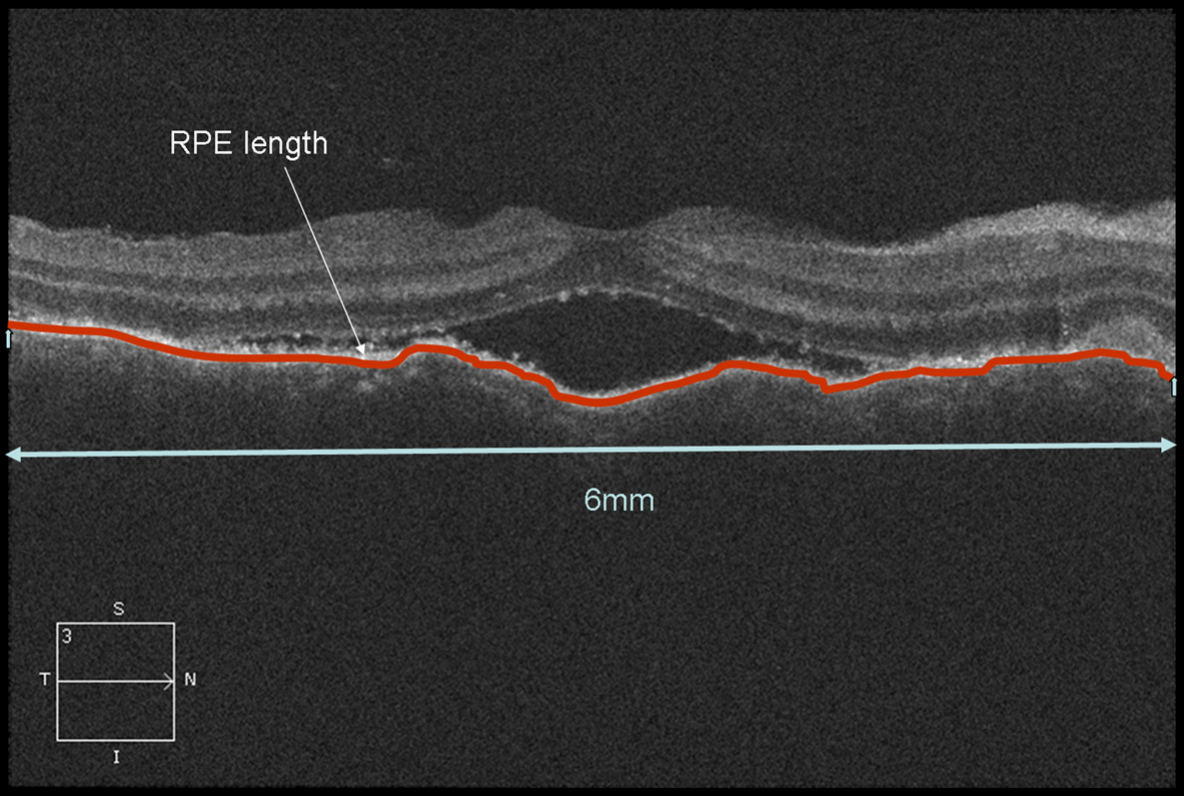
**Calculation of Retinal pigment epithelium undulation index.** The RPE undulation index, defined as (Lh + Lv)/12 000, where Lh and Lv are the RPE line length in the 6 mm foveal-centered horizontal and vertical OCT scans, respectively (red line).

SD-OCT examinations were performed with Cirrus HD-OCT (Carl Zeiss, Germany) using the macular cube 512 × 128 acquisition protocol. OCT images were evaluated by two independent readers (D.L. and Z.Z) who were masked to the clinical diagnosis. For the purpose of this study, RPE fold was defined by at least two sets of peaks and troughs of RPE [15] (Figure 1a). Fluctuation of internal limiting membrane (ILM) was defined as at least two sets of peaks and troughs at the level of ILM (Figure 1a). Subretinal fluid on OCT was defined as homogeneous hypo-reflective space between neurosensory retina and RPE (Figure 1) [16]. Pigment epithelium detachment (PED) was defined as a dome-shaped elevation of the RPE typically seen overlying a homogeneously hypo-reflective space, bound inferiorly by a visible Bruch’s membrane (Figure 1e) [17]. Subretinal septa was defined as a highly reflective line separated from neurosensory retina and continuous with the junction of the photoreceptor inner and outer segments (IS/OS) in attached areas of the retina (Figure 1b,c). Bulge of RPE was defined as a small protrusion of the RPE layer (a slight elevation of the RPE without an area of low reflectivity) (Figure 1e). The RPE undulation index was obtained as described by Hosoda et al. [18]. It was defined as RPE line length to the total scan length ratio on a foveal-centered scan in the SD OCT image (Figure 2). The thickness of fovea was measured as the distance between ILM and the inner border of the line RPE at the central fovea, manually using the caliper in the Cirrus HD-OCT software (version 4.5.1.11). The RPE length measurements were processed by a single operator (D.L.) using ImageJ software (National Institutes of Health, Bethesda, Maryland, USA; available at http://rsb.info.nih.gov/ij/index.html).

### Statistical analysis

Statistical analyses were performed using SPSS for Windows (version 17.0; SPSS, Inc., Chicago, IL). Kappa coefficients of concordance were used to evaluate the agreement between the two readers. The demographic and OCT characteristics were compared between acute VKH disease and acute CSCR. Student’s t tests were performed to compare continuous variables and chi-square test or Fisher’s exact test was performed to compare dichotomous variables between the two diseases. P values of less than 0.05 were considered statistically significant.

## Results

Overall, 3 eyes with VKH syndrome were excluded due to inadequate signal strength. Finally 65 eyes of 34 patients with acute VKH disease and 52 eyes of 52 patients with acute CSCR patients were included in this study. Table 1 demonstrates the demographic information including age and gender in acute VKH disease and acute CSCR. There was a male predominance in the CSCR group, but not in the VKH disease group (90% in CSCR vs. 50% in VKH, p < 0.0001, chi square test). There was no significant difference in patients’ age between the two groups (p = 0.93, student’s t test). BCVA was significantly worse in VKH than in CSCR group (0.84 ± 0.47 vs. 0.33 ± 0.23 LogMAR, p < 0.001, student’s t test, Table 2). Eyes with acute VKH showed an increased RPE undulation index compared to acute CSCR (1.0667 ± 0.0509 vs. 1.0177 ± 0.0023, p = 0.003, student’s t test, Table 2). The kappa coefficients of inter-observer agreement of each OCT features were greater than 0.8 (Table 3).

**Table 1:** Demographic data in patients with VKH disease and CSCR

**Variable**	**VKH**	**CSCR**	**P value**	**Statistical method**
N (people)	34	52		
Age, y (mean ± SD)	40.5 ± 14.4	40.7 ± 7.4	0.93	Student’s t test
(range)	(18–74)	(24–66)		
Gender (male: female)	17:17	45: 7	<0.001	Chi-square test
Time from onset, day			<0.001	Student’s t test
(Mean ± SD)	6.0 ± 2.5	11 ± 6.7		
(Range)	(3–14)	(4–28)		

VKH = Vogt-Koyanagi-Harada; CSCR = central serous chorioretinopathy; SD: standard deviation.

**Table 2:** Comparison of OCT features between acute VKH and acute CSCR

**OCT characteristic**	**VKH**	**CSCR**	**P value**	**Statistical method**
N (eyes)	65	52		
BCVA (LogMAR)^a^	0.84 ± 0.47	0.33 ± 0.23	<0.001	Student’s t test
Foveal thickness (μm)^a^	746.7 ± 423.8	444.9 ± 158.8	<0.001	Student’s t test
Subretinal fluid	65/65 (100%)	52/52 (100%)	1	Fisher’s exact test
Folds of RPE	44/65 (67.7%)	0/52 (0%)	<0.001	Fisher’s exact test
Fluctuation of ILM	34/65 (52.3%)	0/52 (0%)	<0.001	Fisher’s exact test
Subretinal septa	55/65 (84.6%)	0/52 (0%)	<0.001	Fisher’s exact test
PED	2/65 (3.1%)	23/52 (44.2%)	<0.001	Fisher’s exact test
Bulge of RPE	0/65 (0%)	21/52 (40.4%)	<0.001	Fisher’s exact test
RPE length index				
(mean ± SD)	1.0667 ± 0.0509	1.0177 ± 0.0023	=0.003	Student’s t test

OCT = Optical coherence tomography; VKH = Vogt-Koyanagi-Harada; CSCR = central serous chorioretinopathy; BCVA = best corrected visual acuity; LogMAR = logarithm of the minimum angle of resolution; PED = pigment epithelium detachment; RPE = retinal pigment epithelium; ILM = internal limiting membrane.

^a^BCVA and thickness was denoted as mean ± standard deviation.

**Table 3:** Kappa coefficients of concordance between two independent readers on each OCT feature

**OCT character**	**Kappa**
Subretinal fluid	1
Folds of RPE	0.928
Fluctuation of ILM	0.938
Subretinal septa	0.83
PED	0.975
Bulge of RPE	0.883

OCT = Optical coherence tomography; PED = pigment epithelium detachment; RPE = retinal pigment epithelium; ILM = internal limiting membrane.

Figure 1 illustrates the SD-OCT features of acute VKH disease and acute CSCR. Comparison of the OCT characteristics between eyes with acute VKH disease and acute CSCR is shown in Table 3. Thickness of fovea was significantly greater in acute VKH disease (747.7 ± 423.8 μm vs. 444.9 ± 158.8 μm, p < 0.001, student’s t test). All eyes with acute VKH and CSCR had subretinal fluid. Folds of RPE, fluctuation of ILM and subretinal septa were seen in 67.7%, 52.3% and 84.6% eyes with acute VKH disease respectively, but not in eyes with acute CSCR (all p < 0.0001, Fisher’s exact test). However, PED was significantly more common in acute CSCR eyes compared to acute VKH eyes (Figure 1c, 44.2% versus 3.1%, p < 0.001, Fisher’s exact test). The bulge of RPE was present in 40.4% of eyes with CSCR patients but not in eyes with acute VKH disease (p < 0.001, Fisher’s exact test).

## Discussion

In this retrospective study, we compared the tomographic features of acute VKH disease and acute CSCR on SD-OCT. Our results showed that subretinal fluid was present in both diseases. The thickness of fovea and RPE undulation index were significantly greater in VKH compared to that in CSCR. The folds of RPE, fluctuation of ILM and subretinal septa were only present in VKH. On the other hand, CSCR was notable for having significantly more PED and bulge of RPE.

Although the exact pathophysiology of VKH disease and CSCR remains not fully understood, both diseases share common pathogenesis on dysfunction of RPE and choroid. Primary pathology of CSCR is thought to begin with disruption of the choroidal circulation [19]. The RPE is then decompensated and allows exudate from the choroidal vasculature to pass into the subretinal space [20-22]. Various morphologic changes such as PED, bulge of RPE, and subretinal fluid in eyes with CSCR were reported using SD-OCT [23-26]. It was presumed that PED and bulge in the RPE layer may be associated with leakage points in active CSCR. A previous study [27] described retinal pigment epithelial hyperplasia in CSCR. With en face OCT the authors identified this feature in 31% eyes of the CSCR They also found that retinal pigment epithelial hyperplasia was often located at the leaking points observed on FFA. In the current study, the incidence of bulge of RPE (21/52, 40.4%) in acute CSCR cases was similar to those reported in another study (35% and 31%) [24]. This may indicate that bulge of RPE is probably the same as retinal pigment epithelial hyperplasia. The incidence of PED (23/52, 44.2%) was a little lower than that reported in the literature (61%) [24]. Small PEDs may be missed because we only scan the macular region in our subjects, while another previous study [24] applied SD-OCT to detect the RPE changes corresponding precisely to the leakage points on FFA around the whole retina.

Although PED was a less common feature in acute VKH disease [3,28], patients with VKH disease demonstrated significant distortions of the RPE layer on their OCT images. Previous studies have reported folds of the RPE on OCT in eyes with VKH disease [29,30]. Gupta et al. [30] reported that the undulations were observed as peaks and troughs in RPE layer. In current study, we found 44/65 (67.7%) eyes of acute VKH disease had folds of the RPE and the incidence was similar to other literature reports (71.4%) [15]. The RPE undulation index was first described by Hosoda et al. [18]. It can provide quantitative measurement of RPE morphological changes. In the current study, we identified that the distortion of RPE layer in acute VKH was significantly severe than acute CSCR. This may be due to choroidal congestion caused by the infiltration of inflammatory cells. As the retina is softer than the sclera, the RPE could manifest as folds [15].

The subretinal septa was found in 55/65 (84.6%) acute VKH disease eyes but not in acute CSCR eyes. With high resolution SD-OCT, a membranous structure was detected in the floor of cystoid spaces. It was suggested that the membranous structure was a portion of the outer segment (OS) layer that had become separated from the inner segment (IS) layer by cystoid spaces [31]. In our study, the OCT imaging showed that the septa and the cysts were all below the line representing the ELM, the septa was a part of outer segment layer which detached from inner segment (Figure 1b,c). This finding suggests that edema of the photoreceptors may be caused by acute inflammation at choroid [32]. These results also can explain why the visual acuity of acute VKH disease patients was worse than those of acute CSCR patients (0.84 vs. 0.33, LogMAR).

We identified fluctuation of ILM in 34 of 65 eyes (52.3%) on the SD-OCT images of acuteVKH disease patients but none in CSCR patients. To the best of our knowledge, there was no previous report of this characteristic in literature. The mechanism of ILM fluctuation remains unknown. There may be two possible explanations. First, the inflammatory cells infiltrated in vitreous may cause local constriction of ILM. Second, the inflammation may cause diffuse but uneven edema of retina and choroid. The retina may bulge inside in some location and lead to folding of ILM.

Acute VKH disease and acute CSCR share some common clinical manifestation such as the presence of subretinal fluid. The distinguishing features of VKH disease and CSCR on SD-OCT can help us differentiate these two disorders in clinical practice. Furthermore, CSCR is a possible complication of corticosteroids treatment in VKH disease. The morphological changes on OCT could possibly help in differentiating a new recurrence of VKH from CSCR as a side effect of corticosteroids treatment. On the other hand, both VKH disease and CSCR are caused by disorder of choroid and RPE. The different features of VKH and CSCR on SD-OCT may provide insight into the difference of pathogenesis between these two diseases.

We recognize some limitations of our study. First, it is a retrospective study and there may be bias in patient selection. A prospective study including more subjects is needed to confirm our results. Second, there is only one time point in our study. Further studies with longitudinal follow up will provide more insight into the pathogenesis of VKH. Third, the analysis of SD-OCT characteristics is qualitative. Further studies with quantitative image analysis will help us better characterize the SD-OCT images of VKH disease and CSCR. Finally, the images acquired in this VKH and CSCR cohorts were not under the enhanced depth-imaging model. So the image quality did not allow us to evaluate the features of choroid in most of the VKH cases.

## Conclusions

In summary, our study found that on SD-OCT, acute VKH disease and acute CSCR have both similar as well as different features. Subretinal fluid was present in both diseases. The folds of RPE, fluctuations of ILM, and subretinal septa were seen only VKH. RPE bulge was seen only in CSCR. PED was more common in CSCR than in VKH. SD-OCT provides useful information to differentiate CSCR and VKH. It may suggest different pathophysiology of these two diseases.

## Abbreviations

VKH: Vogt-Koyanagi-Harada; CSCR: Central serous chorioretinopathy; SD-OCT: Spectral domain optical coherence tomography; RPE: Retinal pigment epithelial; ILM: Internal limiting membrane; PED: Pigment epithelium detachment; FFA: Fundus fluorescein angiography; BCVA: Best-corrected visual acuity; LogMAR: Logarithm of the minimal angle of resolution; IS/OS: Inner and outer segments.

## Competing interests

The authors declare that they have no competing interests.

## Authors’ contributions

HC designed and supervised the study; DL drafted the manuscript; WC, HH, LC and GZ collected the data; DL, HC, LC and ZZ analyzed the data and helped to draft the manuscript. All authors read and approved the final manuscript.

## Pre-publication history

The pre-publication history for this paper can be accessed here:

http://www.biomedcentral.com/1471-2415/14/87/prepub

## References

[B1] Read RW Holland GN Rao NA Tabbara KF Ohno S Arellanes-Garcia L Pivetti-Pezzi P Tessler HH Usui M Revised diagnostic criteria for Vogt-Koyanagi-Harada disease: report of an international committee on nomenclature Am J Ophthalmol 2001 131 5 647 652 10.1016/S0002-9394(01)00925-4 11336942

[B2] Fang W Yang P Vogt-koyanagi-harada syndrome Curr Eye Res 2008 33 7 517 523 10.1080/02713680802233968 18600484

[B3] Rubsamen PE Gass JD Vogt-Koyanagi-Harada syndrome. clinical course, therapy, and long-term visual outcome Arch Ophthalmol 1991 109 5 682 687 10.1001/archopht.1991.01080050096037 2025171

[B4] Yang CM Lin CP Bullous retinal detachment in a patient with central serous chorioretinopathy J Formos Med Assoc 1998 97 10 711 714 9830282

[B5] Carvalho-Recchia CA Yannuzzi LA Negrao S Spaide RF Freund KB Rodriguez-Coleman H Lenharo M Iida T Corticosteroids and central serous chorioretinopathy Ophthalmology 2002 109 10 1834 1837 10.1016/S0161-6420(02)01117-X 12359603

[B6] Bowie EM Folk JC Barnes CH Corticosteroids, central serous chorioretinopathy, and neurocysticercosis Arch Ophthalmol 2004 122 2 281 283 10.1001/archopht.122.2.281 14769610

[B7] Kang JE Kim HJ Boo HD Kim HK Lee JH Surgical management of bilateral exudative retinal detachment associated with central serous chorioretinopathy Korean J Ophthalmol 2006 20 2 131 138 10.3341/kjo.2006.20.2.131 16892652 PMC2908829

[B8] Arantes TE Garcia CR Rossi MR Muccioli C Spectral domain optical coherence tomography and angiographic findings in central serous chorioretinopathy complicated by bilateral nonrhegmatogenous retinal detachment associated with systemic corticosteroids Ocul Immunol Inflamm 2009 17 5 316 318 10.3109/09273940903030888 19831561

[B9] Kunavisarut P Pathanapitoon K van Schooneveld M Rothova A Chronic central serous chorioretinopathy associated with serous retinal detachment in a series of Asian patients Ocul Immunol Inflamm 2009 17 4 269 277 10.1080/09273940802702579 19657982

[B10] Read RW Yu F Accorinti M Bodaghi B Chee SP Fardeau C Goto H Holland GN Kawashima H Kojima E Lehoang P Lemaitre C Okada AA Pivetti-Pezzi P Secchi A See RF Tabbara KF Usui M Rao NA Evaluation of the effect on outcomes of the route of administration of corticosteroids in acute Vogt-Koyanagi-Harada disease Am J Ophthalmol 2006 142 1 119 124 10.1016/j.ajo.2006.02.049 16815259

[B11] Sasamoto Y Ohno S Matsuda H Studies on corticosteroid therapy in Vogt-Koyanagi-Harada disease Ophthalmologica 1990 201 3 162 167 10.1159/000310145 2089358

[B12] Sakamoto A Hangai M Yoshimura N Spectral-domain optical coherence tomography with multiple B-scan averaging for enhanced imaging of retinal diseases Ophthalmology 2008 115 6 1071 1078 e1077 10.1016/j.ophtha.2007.09.001 18061270

[B13] Srinivasan VJ Wojtkowski M Witkin AJ Duker JS Ko TH Carvalho M Schuman JS Kowalczyk A Fujimoto JG High-definition and 3-dimensional imaging of macular pathologies with high-speed ultrahigh-resolution optical coherence tomography Ophthalmology 2006 113 11 2054 e2051-2014 10.1016/j.ophtha.2006.05.046 17074565 PMC1939823

[B14] AE M Symposium: macular diseases, clinical manifestations Trans Am Acad Ophthalmol Otolaryngol 1965 69 605 613 14345766

[B15] Kato Y Yamamoto Y Tabuchi H Fukushima A Retinal pigment epithelium folds as a diagnostic finding of Vogt-Koyanagi-Harada disease Jpn J Ophthalmol 2013 57 1 90 94 10.1007/s10384-012-0212-x 23149670

[B16] Keane PA Aghaian E Ouyang Y Chong LP Sadda SR Acute severe visual decrease after photodynamic therapy with verteporfin: spectral-domain OCT features Ophthalmic Surg Lasers Imaging 2010 41 Suppl S85 88 10.3928/15428877-20101031-10 21117609

[B17] Bloom SM Singal IP The outer Bruch membrane layer: a previously undescribed spectral-domain optical coherence tomography finding Retina 2011 31 2 316 323 10.1097/IAE.0b013e3181ed8c9a 20890240

[B18] Hosoda Y Uji A Hangai M Morooka S Nishijima K Yoshimura N Relationship between retinal lesions and inward choroidal bulging in Vogt-Koyanagi-Harada disease Am J Ophthalmol 2014 157 5 1056 1063 10.1016/j.ajo.2014.01.015 24491415

[B19] Yannuzzi LA Central serous chorioretinopathy: a personal perspective Am J Ophthalmol 2010 149 3 361 363 10.1016/j.ajo.2009.11.017 20172062

[B20] Holz FG Spaide RF Medical retina. Berlin 2005 New York: Springer

[B21] Wang M Munch IC Hasler PW Prunte C Larsen M Central serous chorioretinopathy Acta Ophthalmol 2008 86 2 126 145 10.1111/j.1600-0420.2007.00889.x 17662099

[B22] Iida T Kishi S Hagimura N Shimizu K Persistent and bilateral choroidal vascular abnormalities in central serous chorioretinopathy Retina 1999 19 6 508 512 10.1097/00006982-199911000-00005 10606450

[B23] Mitarai K Gomi F Tano Y Three-dimensional optical coherence tomographic findings in central serous chorioretinopathy Graefes Arch Clin Exp Ophthalmol 2006 244 11 1415 1420 10.1007/s00417-006-0277-7 16596405

[B24] Fujimoto H Gomi F Wakabayashi T Sawa M Tsujikawa M Tano Y Morphologic changes in acute central serous chorioretinopathy evaluated by fourier-domain optical coherence tomography Ophthalmol 2008 115 9 1494 1500 1500 e1491-1492 10.1016/j.ophtha.2008.01.021 18394706

[B25] Kim HC Cho WB Chung H Morphologic changes in acute central serous chorioretinopathy using spectral domain optical coherence tomography Korean J Ophthalmol 2012 26 5 347 354 10.3341/kjo.2012.26.5.347 23060721 PMC3464318

[B26] Shin YU Lee BR Retro-mode Imaging for retinal pigment epithelium alterations in central serous chorioretinopathy Am J Ophthalmol 2012 154 1 155 163 e154 10.1016/j.ajo.2012.01.023 22503695

[B27] Lehmann M Wolff B Vasseur V Martinet V Manasseh N Sahel JA Mauget-Faysse M Retinal and choroidal changes observed with ‘En face’ enhanced-depth imaging OCT in central serous chorioretinopathy Br J Ophthalmol 2013 97 9 1181 1186 10.1136/bjophthalmol-2012-302974 23823080

[B28] Moorthy RS Inomata H Rao NA Vogt-Koyanagi-Harada syndrome Surv Ophthalmol 1995 39 4 265 292 10.1016/S0039-6257(05)80105-5 7725227

[B29] Wu W Wen F Huang S Luo G Wu D Choroidal folds in Vogt-Koyanagi-Harada disease Am J Ophthalmol 2007 143 5 900 901 10.1016/j.ajo.2006.11.050 17452188

[B30] Gupta V Gupta A Gupta P Sharma A Spectral-domain cirrus optical coherence tomography of choroidal striations seen in the acute stage of Vogt-Koyanagi-Harada disease Am J Ophthalmol 2009 147 1 148 153 e142 10.1016/j.ajo.2008.07.028 18834577

[B31] Ishihara K Hangai M Kita M Yoshimura N Acute Vogt-Koyanagi-Harada disease in enhanced spectral-domain optical coherence tomography Ophthalmology 2009 116 9 1799 1807 10.1016/j.ophtha.2009.04.002 19643489

[B32] Lee JE Park SW Lee JK Choi HY Oum BS Kim HW Edema of the photoreceptor layer in Vogt-Koyanagi-Harada disease observed using high-resolution optical coherence tomography Korean J Ophthalmol 2009 23 2 74 79 10.3341/kjo.2009.23.2.74 19568354 PMC2694296

